# What is the awareness of rare diseases among medical students? A survey in Bulgaria

**DOI:** 10.1186/s13023-023-02820-4

**Published:** 2023-07-25

**Authors:** Eleonora Hristova-Atanasova, Georgi Iskrov, Ivan Atanasov, Atilla Genc, Rumen Stefanov

**Affiliations:** 1 Department of Social Medicine and Public Health, Faculty of Public Health, Medical University of Plovdiv, Plovdiv, Bulgaria; 2 Institute for Rare Diseases, Plovdiv, Bulgaria; 3 Department of Pediatrics, Pulmed University Hospital, Plovdiv, Bulgaria; 4 Faculty of Medicine, Medical University of Plovdiv, Plovdiv, Bulgaria

**Keywords:** Rare diseases (RDs), Orphan drugs, Student research, Bulgaria, Medical education, Orphan drugs, Primary prevention

## Abstract

**Background:**

Rare diseases (RDs) are life-threatening or chronically debilitating and offer a high level of complexity. The aim of this study is to assess medical students’ knowledge and awareness of RDs as well as their perceptions of potential measures to boost training in RDs. The cross-sectional survey was conducted at the Medical University of Plovdiv, Bulgaria, in 2019. The questionnaire contained 12 questions, divided into three main categories: (1) sociodemographic profile; (2) knowledge and awareness of RDs; and (3) attitudes about potential measures to improve training in RDs.

**Results:**

A total of 1189 medical students completed the survey with an overall response rate of 56.4%. Only 13% of participants knew the correct definition of RDs, and a low overall level of awareness was found with regard to orphan drugs (20.3%) and genetic counselling and testing (0.5%). Respondents believed that society as a whole was largely unaware of RDs as a major public health issue. Students suggested elective courses, and invited lectures by RDs experts, and participation in research projects as the most preferred measures to improve undergraduate training.

**Conclusions:**

It is crucial to address the gaps in medical students’ knowledge and awareness of RDs. University curricula should consider incorporating different RDs training modalities. It is essential to encourage various stakeholders to play a more proactive role and to collaborate in these activities. Involvement of patient organisations and advocacy groups might enhance students’ knowledge of the challenges faced by people with RDs. Not least, the media should be partners in this important endeavour as well.

## Background

A disease is considered rare in the European Union (EU) if it affects no more than 5 in 10,000 people. Although the prevalence of a single rare condition is low, it is believed that there are between 5,000 and 8,000 rare diseases (RDs) currently identified. Combined, they affect about 6% of the general population, accounting for more than 29 million individuals in the EU Member States [1]. RDs are life-threatening or chronically debilitating and offer a high level of complexity. All these factors led the Council of the EU to acknowledge RDs as a serious “threat to the health of EU citizens” in 2009 [2, 3].

Over the years, the EU has strongly promoted the adoption of national plans and strategies for RDs as a main policy tool to integrate various initiatives at local, regional, and national levels for a comprehensive approach to this public health problem [1, 2, 4–12]. Bulgaria was the second EU member state (after France) and the first Eastern European country to implement a national plan for RDs (2009–2014) [13]. It stated nine main priorities, including the creation of a national registry, the expansion of newborn screening, and improved diagnostics for RDs. Nevertheless, most of these goals were not achieved. Adoption of a second plan to carry on these activities was discussed but never materialised.

Bulgaria’s national registry for RDs was officially launched in 2017. The National Center for Public Health and Analysis is in charge of hosting and managing it. The registry collects epidemiological data for a predefined list of RDs only from officially designated centers of expertise [14, 15]. Therefore, its scope is limited by default. Furthermore, to this date, there are no publicly available reports or publications coming from the registry.

Almost concurrently, Bulgaria started designating RDs centres of expertise in light of the national transposition of Directive 2011/24/EU on the application of patients’ rights in cross-border healthcare. Currently, there are 24 such entities, with all but three located in Sofia [16]. However, they do not cover all 24 therapeutic areas as defined by the European Reference Networks (ERN) for RDs [3, 14, 15]. This means that Bulgarian medical specialists are unable to fully participate in and maximise the added value of the available opportunities for RDs training and the transfer of knowledge at EU level.

Contrary to most chronic diseases, medical expertise in RDs is scarce, and options for therapeutic management are rather limited [17–23]. RDs are given less attention in undergraduate medical education. The Association of American Medical Colleges (AAMC) has noted that there is a critical need for education that focuses on the identification, comprehension, and care of unusual patients, particularly during medical school training [24, 25]. Most of the undergraduate training in genetics and RDs generally takes place in the preclinical years of study [25, 26]. In this context, a study by Haspel et al. and the Canadian Organisation for Rare Disorders [27] further emphasised the continuous necessity for greater integration of RDs teaching into undergraduate medical education in clinical settings.

The absence of specific health policies for RDs as well as a lack of clinical expertise and experience result in delayed diagnosis and limited access to health care for these patients. It could take between 3 and 14 years from the onset of symptoms to a final diagnosis for certain RDs. Social consequences like isolation and discrimination further severe the impact of RDs on the patients affected and their families [28–32]. All this considered, undergraduate medical education appears to be a major pillar for a sustainable, long-term improvement of care for RDs patients and their families.

Undergraduate medical training in Bulgaria falls into one of the so-called regulated educational fields. This area of professional instruction is formally governed by the Council of Ministers’ Ordinance on uniform state requirements for acquiring higher education in the specialties of medicine and dental medicine for the master’s degree [33]. This document defines an official list of academic courses and honoraria. Nevertheless, it is a purely quantitative framework without any mandatory syllabi. Medical universities are autonomous, and they have the prerogative over the organisation, structure, and contents of undergraduate medical courses. For the courses of medical genetics and social medicine, which are particularly relevant for the undergraduate targeted training on rare diseases, it often means that these subjects are usually positioned in the earlier stages of education. As a consequence, the connection between the theoretical training in medical genetics and social medicine on the one hand and the clinical practice on the other is commonly broken. Medical students fail to translate their conceptual knowledge into routine patterns of clinical skills.

To this point, there has been limited research on the role of RDs in undergraduate medical training in Bulgaria. Little is known about the attitudes of local medical students towards RDs and its health and social impact. The aim of the study is to assess medical students’ knowledge and awareness of RDs as well as their perception of potential measures to boost training in RDs.

## Materials and methods

### Study design

This cross-sectional survey was conducted at the Medical University of Plovdiv, Bulgaria, in 2019. The university has undergraduate programmes in medicine with instruction offered in Bulgarian or English (thus, the language of instruction is the main independent variable for comparison). Students attend the same Faculty of Medicine and follow the same academic training programmes.

### Study participants and settings

There were a total of 2107 medical students at the Medical University of Plovdiv in 2019. No specific sampling technique was applied, as all current students at the time of the study were invited to participate in the survey. A direct questionnaire to complete was provided during the regular lectures and practical classes.

### Research tool

The study used an updated version of the original questionnaire that Stefanov et al. developed in 2008 [34]. The survey tool was translated into English and validated with a group of 30 students (Cronbach’s alpha = 0.81). The questionnaire contained 12 questions (Table 1), divided into three main categories: (1) sociodemographic profile; (2) knowledge and awareness of RDs; and (3) attitudes about potential measures to improve training in RDs.

**Table 1:** Summary of the sections and questions

Survey Category	Questions
Sociodemographic Profile	1. Gender
	2. Age
	3. Year of study
Knowledge and Awareness of RDs	4. Definition of RDs
	5. Identification of RDs (yes or no)
	6. Number of RDs patients in Bulgaria
	7. Definition of orphan drugs
	8. Primary prevention of RDs
	9. Challenges for RDs patients and their families
Potential measures to improve training in RDs	10. Role and place of RDs in undergraduate medical training curriculum
	11. Measures to improve undergraduate medical training curriculum on RDs
	12. Public awareness and support

### Data collection

During the regular lectures and practical classes, printed questionnaires were distributed for completion. All present medical students were invited to participate in the survey. Participation was voluntary, and no incentives were offered. The authors supervised the data collection process.

### Ethics approval

Approval by an ethics committee was not required for this study. The survey was sociological from a methodological point of view, with no clinical research. No personal data were saved or analysed.

### Data analysis

Descriptive statistics were applied. Comparisons were made between different demographic variables to determine if they were associated with specific outcomes. Analyses specifically focused on the differences between medical students trained in Bulgarian or English. Statistical significance was considered if the p-value was less than 0.05. Data were analysed using SPSS (version 26.0; SPSS, Inc., Chicago, IL, USA).

Partially completed questionnaires were retained and analysed for better representation of the target population.

## Results

### Sociodemographic profile of the respondents

A total of 1189 medical students completed the survey, with an overall response rate of 56.4%. There were 627 students (47.3%) trained in Bulgarian and 562 (52.7%) trained in English (Table 2). This split was used for further comparison between the two main groups. Second-year students (27.6%) and third-year students (28.3%) made up the biggest part of the respondents.

**Table 2:** Profile of Survey Respondents

Variables		Instruction in Bulgarian		Instruction in English		χ^2^	p
		n	%	n	%		
Gender	Male	240	38.3	284	50.5	18.06	0.000
	Female	387	61.7	278	49.5		
Age groups	≤ 21	387	63.7	309	56.6	17.44	0.000
	22–24	171	28.1	149	27.3		
	≥ 25	50	8.2	88	16.1		
Year of study	First	101	16.3	101	18.1	15.82	0.007
	Second	155	25.0	170	30.5		
	Third	171	27.5	163	29.2		
	Fourth	117	18.8	83	14.9		
	Fifth	65	10.5	38	6.8		
	Sixth	12	1.9	3	0.5		

### Respondents’ knowledge and awareness of RDs

**Fig. 1:**
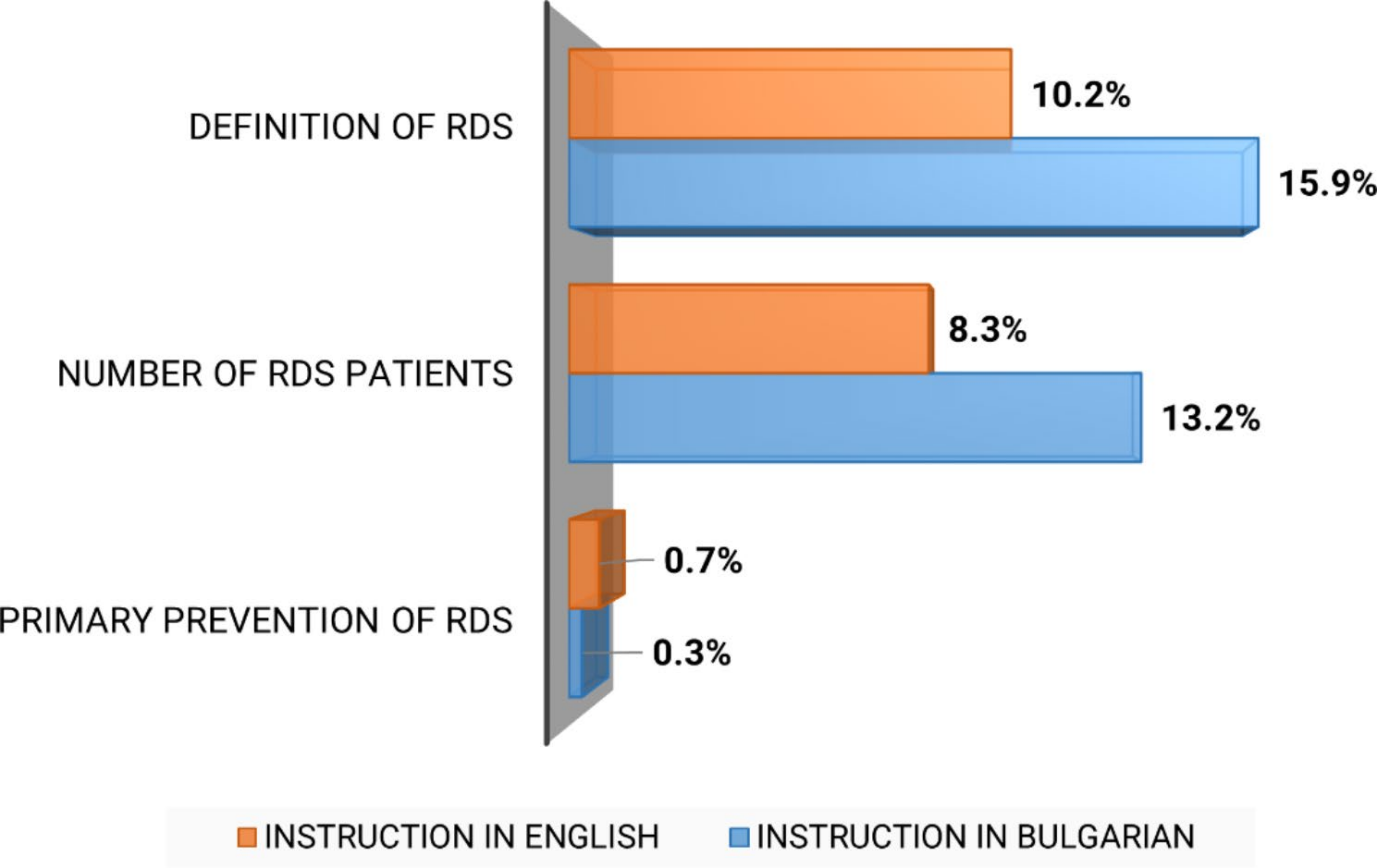
Respondents’ Knowledge and Awareness of RDs (rate of correct responses)

Only 13% (n = 145) of all participants knew the correct definition of RDs (Fig. 1).

**Fig. 2:**
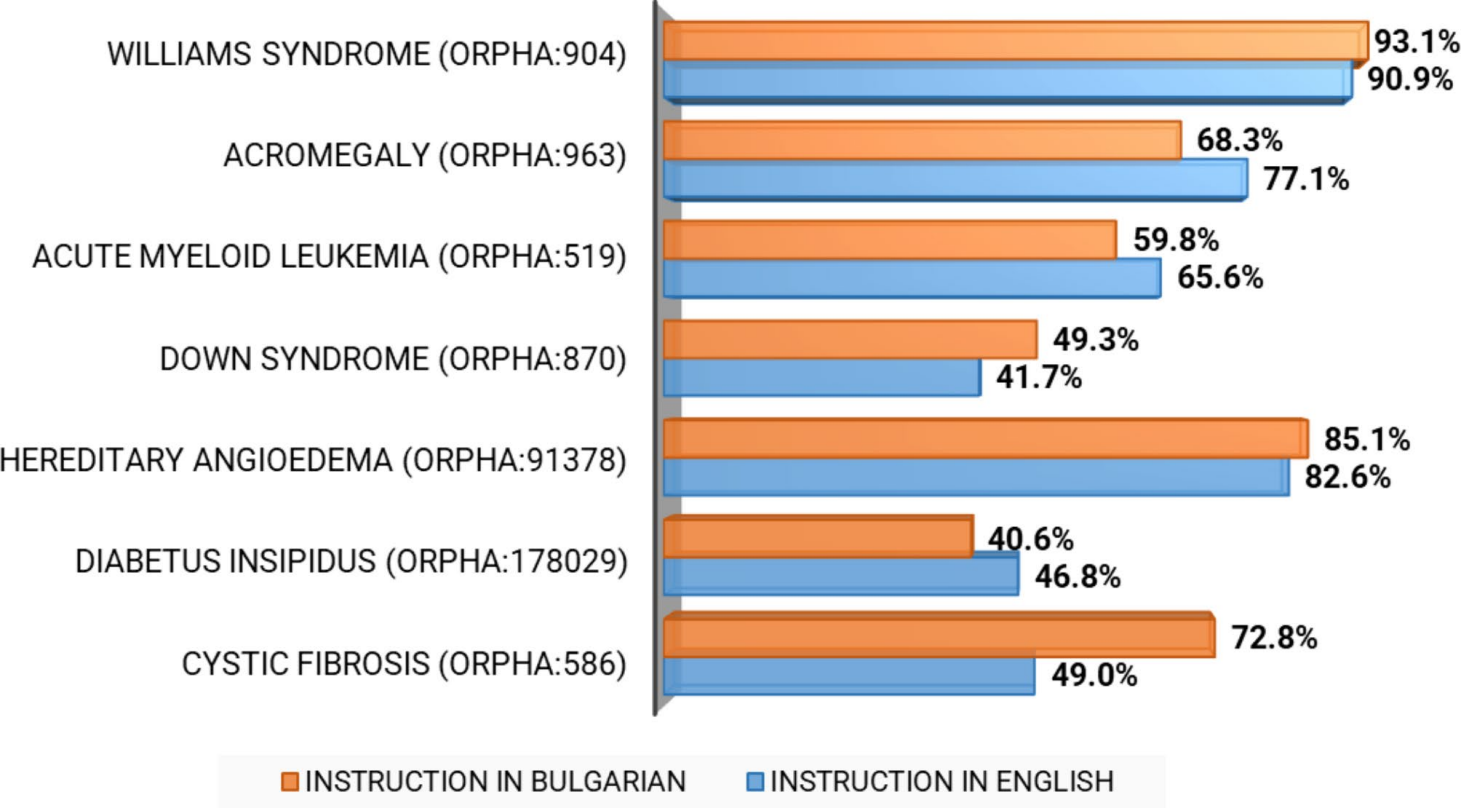
Respondents’ Identification of RDs (rate of correct responses)

Williams syndrome was the most frequently correctly identified RDs (Fig. 2). On the other side, Down syndrome and diabetes insipidus were listed as RDs by less than half of the students. There was a statistically significant difference in the rate of correct identification of RDs between the students trained in Bulgarian and English, with the exception of Williams syndrome (p > 0.05). The biggest gap was observed in the case of cystic fibrosis. By contrast, all students demonstrated at least a 90% correct response rate on the same question about common diseases.

**Fig. 3:**
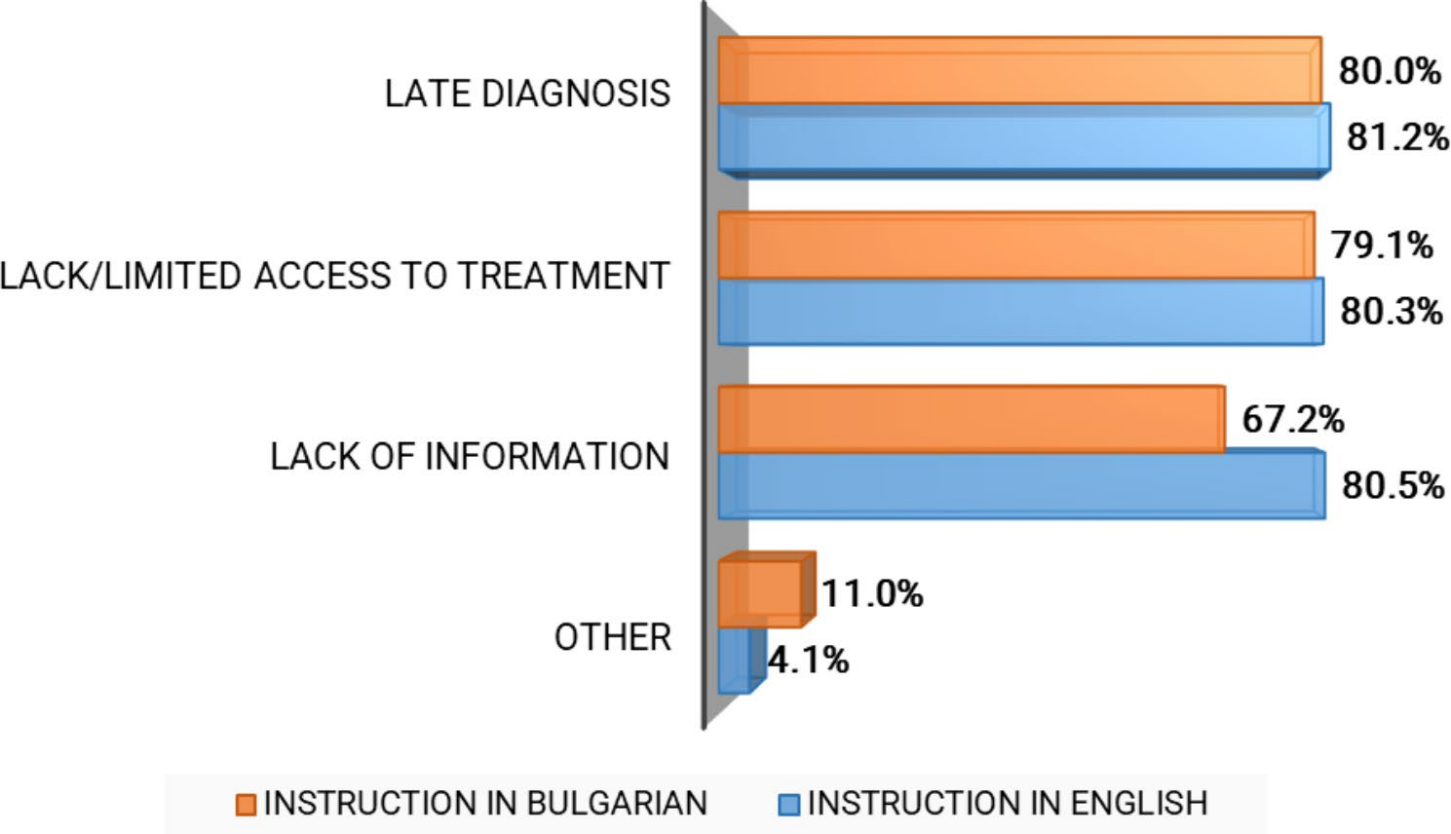
Respondents’ Knowledge and Awareness of the Challenges for RDs patients and their families (rate of agreement)

Few students from both groups correctly stated that there are approximately 450 000 RDs patients in Bulgaria: 8.3% (n = 43) of those trained in English and 13.2% (n = 78) of those trained in Bulgarian (p > 0.05). A similar low overall level of awareness was found with regard to orphan drugs (20.3%, n = 138) and genetic counselling and testing (0.5%, n = 6) (Fig. 1). Late diagnosis, lack of or limited access to therapy, and lack of information were indicated as the most important problems that RDs patients face, with the latter being the only point of disagreement between the two student groups (p = 0.000) (Fig. 3). Respondents believed that society as a whole was largely unaware of RDs as a major public health issue, with only 2.9% (n = 18) of the students trained in Bulgarian and 11.0% (n = 60) of those trained in English stating the opposite (p = 0.000).

### Respodents’ attitudes about potential measures to improve training on RDs

The two groups of students disagreed on the impact of RDs in the current curriculum. While 56.9% (n = 312) of the students trained in English stated that RDs were reflected to a certain extent, only 24.8% (n = 150) of those trained in Bulgarian thought the same (p = 0.000). Both samples cited elective courses in RDs, invited lectures by RDs experts, and participation in RDs research projects as the most preferred measures to improve undergraduate training. Information on the Internet and printed materials about RDs were consistently among the lowest rated approaches, with 12.8% (n = 67) of the students trained in English and 22.5% (n = 141) of those trained in Bulgarian expressing a preference for them (p = 0.000). On the other hand, RDs conferences and workshops were positively assessed by the vast majority of both groups—64.8% (n = 340) of the respondents in English and 82.3% (n = 497) of the respondents in Bulgarian (p = 0.000). Finally, students pointed out the role of the media in raising awareness about RDs in society. The overwhelming majority—84.9% (n = 508) of the students trained in Bulgarian and 75.8% (n = 398) considered that the media should act more to promote the recognition of RDs as a substantial public health priority (p = 0.000).

## Discussion

### Potential educational measures to improve training on RDs

This study confirmed previous findings from other countries that medical students generally lack knowledge and awareness of RDs [30, 31, 35–37]. While there was some disagreement between the two groups of respondents in our research, they largely found common ground on the most important issues and expressed support for similar measures to improve undergraduate training in RDs.

Several previous studies have explored the level of knowledge and awareness of RDs among medical students from different countries. Although direct comparison is not meaningful due to the differences in the studies’ methodology and research context, there is a recurring pattern of low understanding of RDs and their key public health aspects [38–44]. Trainees lack basic knowledge about issues like primary prevention and especially folic acid intake [39–44]. Premarital genetic counselling is one of the most successful tools for the primary prevention of genetic disorders. Therefore, its place and role in medical education should be amplified.

In all the studies conducted so far, medical students acknowledged the need for more concentrated training in RDs by mentioning elective courses in RDs and invited lectures by RDs experts as potential options to boost medical undergraduate education in RDs. Participation in RDs research projects and scientific events is also welcomed by the respondents to those surveys [30, 31, 35–38, 45–48]. In our study, we witnessed comparable gaps in knowledge in both students trained in Bulgarian and in English. Substantial portions of the participants from the two groups could not identify the definitions of RDs and orphan drugs. What is even more alarming is the lack of contrast between students of different years of training, indicating neither improvement nor accumulation of RDs knowledge in the course of the 6-year cycle of undergraduate medical training in Bulgaria. The public health implications of this educational void are great and contribute to delayed access to RDs diagnosis and treatment [38, 47, 49].

### Potential collaborations with stakeholders to improve training on RDs

Acquiring professional medical experience and expertise takes time and resources. Information in the literature about RDs is often scarce. RD-specific guidelines are usually limited only to conditions where medicinal treatment options are available, but those represent a small fraction of all RDs. In this light, collaboration and cooperation with patient organisations and advocacy groups may be highly beneficial. Learning from RDs patients and their families could make the next generation of medical professionals think outside the box. RDs medical training does indeed need an unconventional style and a new perspective [35, 46]. Medical students, as future doctors, are in a unique position to transform the health system and make it more aware of RDs.

Of course, the role of academic staff in this overall process is undeniable [50, 51]. This is why experienced clinical instructors in RDs must be attracted to teaching and promoting this complex topic among medical students. In this context, the current ERN for RDs offers a great opportunity to connect undergraduate medical training with the reality of RDs. Renowned healthcare providers from all EU Member States participate in these virtual networks. ERN are meant to encourage cooperation and the transfer of know-how in the field of RDs healthcare, which by definition is highly specialised and resource-demanding. A symbiosis between ERN and medical universities could generate great added value for all European RDs stakeholders. Another major international tool for dissemination and promotion of knowledge about RDs is the Orphanet portal for RDs and orphan drugs [52–55]. Over the past 25 years of activity, Orphanet has become the leading reference source of information on RDs and has helped shape the RD landscape in Europe and around the world. With the challenges of RDs medical training in mind, there is a strong need to encourage both ERN and Orphanet to do more of this kind of academic partnership to help medical students learn more [56].

Finally, the media and society as a whole should also be more proactive in the case of RDs [10, 57]. RDs are not just a medical problem. Their spillover effects go way beyond the healthcare system and are associated with high levels of socioeconomic burden. From this point of view, engaging young medical doctors from the very early stages of training is essential and could largely help improve RDs diagnosis, treatment, and follow-up. If more medical professionals are aware of and experienced with RDs, patients will have easier access to health care and services. Subsequently, this could mean reduced resource consumption, as delayed diagnosis and difficult access to treatment are well-known factors for worse health outcomes and higher costs [58].

### Limitations

Although this study provides insights about Bulgarian medical students’ knowledge and awareness of RDs as well as their perception of potential measures to boost training in RDs, our findings are limited in several ways. First, despite the relatively large sample size achieved, the survey only targeted students from a single university in Bulgaria. Thus, our research outcomes cannot be directly generalised. Second, there was an overrepresentation of preclinical year students in our study sample. The response rates among fifth- and sixth-year students were low. Nevertheless, the latter could also be considered an indirect indication of a lack of interest and subsequent limited knowledge about RDs. Third, the list of conditions used to identify RDs was taken from the original questionnaire by Stefanov et al. This set cannot be considered representative of RDs knowledge and awareness as a whole. Nonetheless, the list contains different RDs by prevalence and aetiology and offers a good starting point for research. Fourth, it should be noted that the two groups in our study came from very different backgrounds. Students who are trained in Bulgarian are almost exclusively from Bulgaria, while students trained in English come from various countries throughout the world. This, of course, could result in different levels of awareness and perception of RDs depending on the country of origin. Still, the two samples largely overlapped in terms of both knowledge and measures to improve undergraduate training in RDs. It could probably be concluded that RDs are perceived globally as an important void in medical education and must be subjected to targeted actions.

## Conclusions

The survey demonstrated a lack of medical knowledge of RDs as well as a low level of awareness regarding orphan drugs and genetic counselling among students. Respondents highlighted the need for more concentrated training in RDs by offering elective courses and inviting RDs experts as guest lecturers. Students believed that society as a whole was mostly uninformed about RDs as a higher public health concern and that late diagnosis and limited access to therapy were the two most crucial challenges that RDs patients confronted.

It is crucial to address the gaps in medical students’ knowledge and awareness of RDs. University curricula should consider incorporating different RDs training modalities. It is essential to encourage various stakeholders to play a more proactive role and to collaborate in these activities. Involvement of patient organisations and advocacy groups might enhance students’ knowledge of the challenges faced by people with RDs. Not least, the media should be partners in this important endeavour as well.

## Data Availability

The data that support the findings of this study are not openly available due to reasons of sensitivity but are available from the corresponding author upon reasonable request. Data are located in controlled access data storage at the Department of Social Medicine and Public Health.
